# Low-dose ozone application during storage modulates yolk oxidation and enhances hatchability in broiler hatching eggs

**DOI:** 10.1093/jas/skag155

**Published:** 2026-06-01

**Authors:** Sinan Vicil, Fatma Tülin Özbaşer Bulut, Metin Bayraktar, Muazzez Gürgan Eser

**Affiliations:** Faculty of Veterinary Medicine, Department of Biochemistry, Tekirdag Namik Kemal University, Tekirdag, 59030, Turkey; Faculty of Veterinary Medicine, Department of Animal Science, Tekirdag Namik Kemal University, Tekirdag, 59030, Turkey; Faculty of Veterinary Medicine, Department of Animal Science, Tekirdag Namik Kemal University, Tekirdag, 59030, Turkey; Faculty of Arts and Sciences, Department of Biology, Tekirdag Namik Kemal University, Tekirdag, 59030, Turkey

**Keywords:** broiler breeder, egg quality, hatchability, malondialdehyde, ozone, storage

## Abstract

This study evaluated whether low-dose ozone exposure during storage can mitigate the negative effects of long-term storage on egg quality, yolk oxidative status, and hatching outcomes in broiler hatching eggs. A total of 1,350 eggs from 67-wk-old Ross 308 breeders were allocated to a 2 × 3 factorial arrangement of treatments: storage for 0 or 14 d and daily exposure to 0, 4, or 7 ppm ozone for 30 min. Egg quality traits (albumen index, yolk index, Haugh unit, shell thickness and ratios), eggshell microbial load (log_10_ CFU/mL), yolk malondialdehyde (MDA; nmol/mg protein), yolk protein content (mg/mL), and hatchability and embryonic mortality were recorded. Data were analyzed by two-way ANOVA with storage time and ozone dose as fixed effects. Fourteen days of storage reduced albumen index, yolk index, and Haugh unit and increased yolk MDA (5.70 vs. 8.87 nmol/mg protein; *P* < 0.001) and protein content (14.34 vs. 15.24 mg/mL; *P*  < 0.01). Ozone markedly reduced eggshell microbial load in non-stored and stored eggs (eg in stored eggs, 9.14, 3.69, and 1.77 log_10_ CFU/mL at 0, 4, and 7 ppm; *P*  = 0.001). Yolk MDA was greater at 7 ppm than at 0 or 4 ppm at both storage times (*P*  ≤ 0.002), whereas 4 ppm did not differ from 0 ppm. For eggs stored, 0 d, 4 ppm increased hatchability of fertile eggs (96.42%) compared with 0 ppm (91.07%; *P*  = 0.034). After 14 d of storage, 4 ppm resulted in greater hatchability of fertile eggs than 7 ppm (92.92 vs. 80.39%; *P*  = 0.025), whereas values for the 0 ppm group (86.45%) were intermediate and did not differ from either group; 4 ppm also reduced early embryonic mortality compared with 7 ppm (2.65 vs. 11.76%; *P*  = 0.007). In conclusion, low-dose ozone (4 ppm) applied during storage reduced eggshell microbial load and supported embryonic viability and hatchability without increasing yolk lipid peroxidation, whereas higher ozone (7 ppm) increased yolk MDA and early embryonic loss in long-stored eggs.

## Graphical abstract

**Graphical Abstract:**
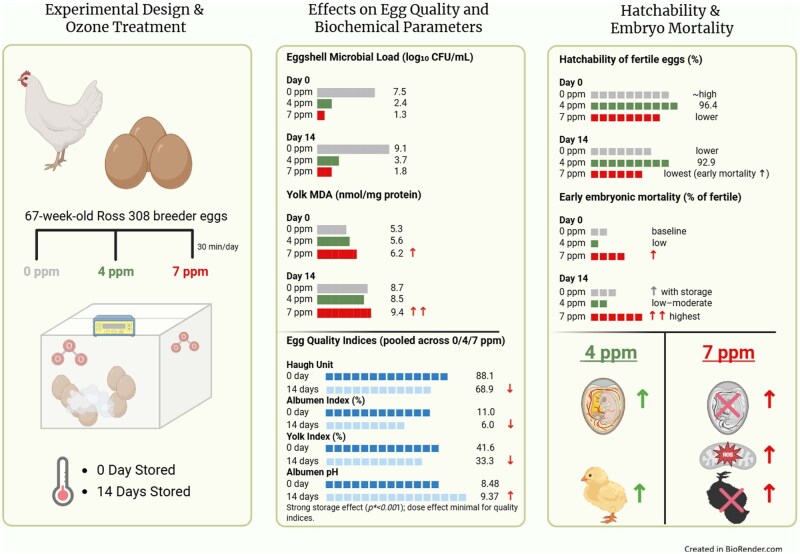

## Teaser

Daily exposure of broiler breeder eggs to low-dose ozone (4 ppm) during storage improved hatchability of fertile eggs and reduced shell microbial load without exacerbating yolk lipid oxidation. In contrast, a higher ozone dose (7 ppm) increased yolk oxidative damage and early embryonic mortality, indicating a narrow safety window for ozone-based egg disinfection.

## Introduction

Storage of hatching eggs is widely used in hatcheries to coordinate hatching, but prolonged storage can impair hatchability and chick quality (Fasenko 2007; Adriaensen et al. 2022). During storage, eggshells carrying natural and environmental microflora may become contaminated, and microorganisms can proliferate and penetrate through pores, deteriorating egg quality and increasing early embryo mortality (Kożuszek et al. 2009). Chemical disinfectants have long been applied to hatching eggs to limit these effects, yet some agents leave toxic residues on the shell, pose carcinogenic risks, or negatively affect shell quality (Sheldon and Brake 1991; Clímaco et al. 2018).

Ozone (O_3_) is a strong oxidant that has gained attention as an alternative sanitizer in the food and poultry industries, where gaseous or aqueous ozone is used for air purification and eggshell decontamination because it rapidly decomposes to oxygen without residues (Christ et al. 2017; Koç and Aygün 2021). However, ozone and its reactive species can also oxidize biological molecules and may affect embryo physiology if the dose is excessive (Cataldo 2003; Giménez et al. 2024). Despite the use of storage and disinfection procedures in broiler hatcheries, there is limited information on how different ozone doses affect eggshell microbial load, egg quality, yolk oxidative status, and hatching outcomes in long-term storage. Therefore, the present study investigated the effects of daily ozone exposure at 0, 4, or 7 ppm during storage on egg quality traits, yolk malondialdehyde (MDA) and protein levels, eggshell microbial load, and hatchability in broiler hatching eggs stored for 0 or 14 d. We hypothesized that low-dose ozone during storage would condition eggs, reducing eggshell microbial load and supporting development and hatchability without increasing yolk lipid peroxidation, whereas a higher dose would increase oxidative damage and impair hatching outcomes in long-stored eggs.

## Materials and methods

### Animal material and experimental design

All procedures involving animals were approved by the Tekirdag Namik Kemal University Animal Experiments Local Ethics Committee (03/07/2025- T2025/2800-5) and were conducted in accordance with national and institutional guidelines for the care and use of animals.

The study was conducted on 1,350 eggs collected daily from 67-wk-old Ross 308 broiler breeder hens raised on a private farm (Pak Poultry Company, Kırklareli, Türkiye). Eggs were randomly assigned to three treatment groups (control, 4 ppm ozone, and 7 ppm ozone). Within each treatment, eggs were further sampled as two subgroups: non-stored (fresh) and stored for 14 d. Thus, a 3 (ozone dose) × 2 (storage duration) factorial experimental design was formed. Three replicates were established per group, with 75 eggs used in each replicate. For each treatment group, a total of 225 eggs (75 eggs × 3 replicates) were allocated. For egg quality analyses, 21 eggs per group (7 eggs per replicate) were randomly selected prior to incubation and used for measurements of external and internal quality traits. Control groups (0 ppm ozone) were not subjected to any disinfection treatment and included both non-stored (fresh), and eggs stored for 14 d and were maintained under identical storage and incubation conditions as the ozone-treated groups.

Exposure of eggs to ozone was carried out in an airtight chamber of 1.5 m³, with environmental conditions set to 20 °C and 75% relative humidity. Ozone in the gas phase was generated using a fan-equipped generator (ATWFS, 220 V–60 g, China). For the non-stored subgroup, a single ozone application was performed immediately before setting; for the 14-d stored subgroup, ozone was applied daily during storage at target concentrations of 4 ppm and 7 ppm for 30 min each. The ozone concentration inside the chamber was measured in real time and maintained at the target value using a digital ozone sensor (ZE27-O3, Winsen, Zhengzhou, China; detection range: 0–10 ppm; resolution: 0.01 ppm). Fertile eggs were stored for 14 d in a dedicated storage room at 15 °C and 75% relative humidity and were turned twice daily.

### Egg quality characteristics

Before setting in the incubator, seven eggs were randomly selected from each replicate and weighed using a digital balance with 0.01 g precision. Egg length and width were measured using a caliper, and the shape index was calculated (Cüneydioğlu et al. 2022; Özbaşer Bulut et al. 2025). The selected eggs were then broken on a glass surface to determine certain external (shape index, egg weight, shell thickness) and internal quality traits (albumen and yolk ratio, albumen and yolk index, Haugh unit). Albumen length, albumen width, and yolk diameter were measured with a digital caliper; albumen height and yolk height were measured with a tripod micrometer. After separating the albumen and yolk, yolk weight was recorded. Albumen weight was calculated as: egg weight − (yolk + shell weight) (Cüneydioğlu et al. 2022). Albumen and yolk percentages were obtained by dividing the respective weights by egg weight and multiplying by 100. Albumen and yolk indices were calculated using formulas reported by previous researchers (Onbaşılar et al. 2011; Cüneydioğlu et al. 2022) as follows:


Albumen index (%)=[albumen height/ ((albumen length+albumen width)/2)]×100

Yolk index (%)=(yolk height/yolk diameter)×100

Albumen height was related to egg weight to determine the Haugh unit (Özbaşer Bulut et al. 2025): *HU = 100 log_10_ (H − 1.7 W^0.37^ + 7.6), where W: egg weight; H: albumen height.*

Albumen pH values were determined individually for each egg sample using a digital pH meter. Shell thickness was measured after washing the broken shells, drying them at room temperature for 24 h, and then taking measurements at three different regions (blunt end, equator, pointed end) using a digital micrometer with 0.001 mm precision. The mean of the three readings was recorded as shell thickness.

The lipid peroxidation level in egg yolk was measured using the MDA Colorimetric Assay Kit, TBA Method (E-BC-K025-M, Elabscience, Wuhan, China), following the manufacturer’s protocol. Briefly, 0.5 g of yolk was homogenized on ice with cold PBS (pH 7.4) at a ratio of 1:9 (w/v). After centrifugation at 10,000 g for 10 min at 4 °C, the supernatant was used. Samples/standards were mixed with kit reagents and incubated at 100 °C for 40 min. After cooling, absorbance was read at 532 nm. MDA concentration was calculated from the calibration curve constructed with kit standards and reported as nmol/mg protein. Total protein concentration in the yolk was determined by the Bradford method using bovine serum albumin as the standard (Bradford reagent; Sigma-Aldrich, St. Louis, MO, USA) and expressed as mg/mL (duplicate measurements).

### Microbiological examination

From each group, the eggshell surface of 10 eggs was swabbed using a cotton swab moistened with sterile PBS and transferred into a test tube containing 5 mL of sterile PBS. Samples were transported to the laboratory as quickly as possible on ice packs. Upon arrival, each suspension was vortexed for 30 s to ensure homogeneous distribution before serial operations under aseptic conditions. For total aerobic bacterial counts, 0.1 mL aliquots were spread onto sterile plate count agar (Merck Co., Whitehouse Station, NJ). Plates were incubated at 37 °C for 24–48 h. After incubation, colonies were counted and recorded as colony-forming units (CFU/mL) (Jones et al. 2002).

### Evaluation of incubation results

Eggs disinfected with two different ozone doses were placed in the incubator (VGS-90, VGS Industry Ltd., Turkey) either immediately (fresh) or after 14 d of storage. During storage, eggs assigned to the treatment groups received ozone gas daily at 4 or 7 ppm for 30 min. Eggs were incubated at 37.2 °C and 58% relative humidity for the first 18 d, and at 36.5 °C and 75% relative humidity for the last 3 d (Leão et al. 2024). Eggs were turned every 2 h during the first 18 d. Unhatched eggs were broken out to determine the timing of embryonic death as early, middle, or late. Late embryonic deaths were evaluated as deaths under the shell and deaths due to malposition (pipping malposition).

Early, middle, and late embryonic mortalities for each group were calculated as the percentage of dead embryos in each period out of the total number of fertile eggs. Based on the hatching results, fertility, hatchability, and chick yield were calculated using the formulas reported by Onbaşılar et al. (2011). Accordingly:


Hatchability of total eggs=(Number of chicks hatched/Number of eggs set)×100

Hatchability of fertile eggs=(Number of chicks hatched/ Number of fertile eggs set)×100

Fertility=(Number of fertile eggs/Number of eggs set)×100

Hatch progression was monitored at 8-h intervals from 432 h of incubation onward, and incubation length was recorded for each chick as the observation interval at which it had fully emerged from the shell. Replicate-tray means were calculated by averaging individual chick values within each tray, and group means (± pooled SEM) reported in Table 2 represent the average of the three replicate-tray values per treatment combination (Yılmaz Dikmen et al. 2025). Although observations were captured at discrete 8-h intervals, averaging across multiple chicks within each replicate tray yielded continuous mean values.

### Statistical analyses

The number of eggs to be used in the study groups was determined by a priori power analysis (α  =  0.05, 1−β  =  0.80) using G*Power 3.1.9.2 (Heinrich-Heine-Universität Düsseldorf, Düsseldorf, Germany) (Faul et al. 2007). All statistical analyses were performed using SPSS version 25.0 (IBM Corp., Armonk, NY, USA). Egg quality traits, yolk MDA, yolk protein concentration, and albumen pH were analyzed using a two-way ANOVA with storage time (0 or 14 d) and ozone dose (0, 4, or 7 ppm) as fixed factors, with the individual egg considered as the experimental unit. Hatching parameters (fertility, hatchability of fertile and total eggs, early, middle, and late embryonic mortality, and incubation length) were calculated per replicate tray (*n* = 3 trays per treatment combination, with 68 eggs set per tray after removal of 7 eggs per tray [21 per group] for pre-incubation quality assessment) and analyzed using two-way ANOVA. Proportional data were subjected to arcsine square-root transformation prior to analysis, whereas incubation length was analyzed without transformation. Storage duration and ozone dose were included as fixed factors, and replicate was considered the experimental unit. Fertility was determined among the 204 eggs set per group (excluding the 21 eggs sampled for pre-incubation quality assessment) by candling on day 7 of incubation. For microbiological analyses, eggshell swabs from 10 eggs per replicate tray were pooled per replicate, yielding three pooled samples per treatment combination; each pooled sample was analyzed separately, and the replicate-level pooled sample was considered the experimental unit (*n* = 3). Total aerobic bacterial counts (log_10_ CFU/mL) were analyzed using two-way ANOVA with storage duration and ozone dose as fixed factors. When significant main effects or interactions were detected (*P* < 0.05), means were compared using Tukey’s HSD test. Pooled standard errors of the mean derived from the ANOVA error term are reported in Tables 1 and 2, and back-transformed means are presented for proportional outcomes to facilitate interpretation. A value of *P* < 0.05 was considered statistically significant (Dawson and Trapp 2001).

**Table 1:** Eggshell microbial load by storage duration and ozone dose, log_10_ CFU/mL.

Storage	Ozone application	Microbial growth, CFU/mL
**Non-stored**	0 ppm	7.54 ± 0.77^a^
	4 ppm	2.40 ± 0.39^b^
	7 ppm	1.25 ± 0.20^c^
**Stored 14 d**	0 ppm	9.14 ± 0.62^d^
	4 ppm	3.69 ± 0.47^e^
	7 ppm	1.77 ± 0.21^f^
***P*-value**		
** Storage**		0.001
** Application**		0.001
** Storage × Application**		0.001

Different letters (a, b, c, d, e, f) indicate statistically significant differences in the same column (*P* < 0.05). SE: Standard Error.

**Table 2:** Hatching results in chicks, %.

Parameters	**D 0**				*P*-values	**D 14**				*P*-values
	0ppm	4ppm	7ppm	SEM		0ppm	4ppm	7ppm	SEM	
**Eggs set (n)**	204	204	204			204	204	204		
**Fertile eggs (n)**	155	155	157			136	160	144		
**Fertility (% of eggs set)**	75.98	75.98	76.96	0.03	0.987	66.67	78.43	70.59	0.03	0.077
**Hatchability of fertile eggs (%)**	91.07^b^	96.42^a^	86.72^b^	2.81	0.034	86.45^a,b^	92.92^a^	80.39^b^	3.62	0.025
**Hatchability of total eggs (%)**	69.38	73.46	66.60	2.16	0.441	57.63^b^	72.01^a^	56.94^b^	4.91	0.007
**Early embryo mortality (%)**	5.36	3.57	7.96	1.27	0.399	3.13^b^	2.65^b^	11.76^a^	2.96	0.007
**Middle embryo mortality (%)**	3.57	0.00	2.65	1.07	0.839	3.13	0.00	2.94	1.01	1.000
**Late embryo mortality (%)**	0.00^b^	0.00^b^	2.70^a^	0.90	0.050	7.30	4.40	4.90	0.90	0.665
**Incubation length (h)**	519.30^a^	511.71^b^	514.42^b^	0.22	0.036	524.14	521.00	525.00	1.00	0.234

Different letters (a, b) in the same column indicate statistically significant differences (*P* < 0.05). SEM: Standard Error. Each replicate tray was considered an experimental unit.

## Results

The effects of storage duration and different ozone doses on the microbial load of the eggshell surface are presented in Table 1. Total aerobic bacterial counts were significantly higher in the non-ozonated control groups compared with both the 4 ppm and 7 ppm ozonated groups (*P* < 0.01). Additionally, although prolonged storage duration increased aerobic bacterial counts, the increase was lower in the ozonated groups than in the control (*P* < 0.01) (Table 1).

When the data obtained for hatching parameters in this study were evaluated, it was observed that ozone applied at different doses significantly affected the hatchability of fertile eggs in both non-stored eggs and eggs stored for 14 d (*P* < 0.05). In both storage durations, the results obtained with the 4 ppm dose (d 0: 96.42%; d 14: 92.92%) were generally higher than those of the other groups (Table 2). Findings related to the hatchability of total eggs were not statistically significant among the d-0 experimental groups, whereas they became significant on d 14 (*P* < 0.01). In the study, the 7 ppm application adversely affected hatch performance, particularly increasing early embryonic mortality (11.76%) (*P* < 0.01), and also caused a significant difference in late embryonic losses at d 0 (*P* < 0.05) (Table 2). Mid-term embryonic mortalities did not differ significantly among groups in either period (*P* > 0.05). Between day 0 and day 14 of storage, a statistically significant decrease in the hatchability of total eggs was observed only in the control group (d 0: 69.38%; day 14: 57.63%), whereas in the 7 ppm group, it also decreased but not significantly (*P* = 0.08). Although numerical differences in fertility were observed among groups, these differences were not statistically significant. Therefore, the observed variation is likely attributable to biological variability among eggs, particularly considering the advanced breeder age (67 wk), rather than a direct effect of ozone treatment (Table 2).

Because fertility is determined at oviposition, it is unlikely to be influenced by post-laying treatments such as storage or ozone exposure. Eggs were randomly assigned to treatment groups prior to any storage or disinfection procedure; therefore, the numerical differences in fertility observed at d 14 (*P* = 0.077) most likely reflect random variation rather than a treatment effect. Consequently, these differences are unlikely to meaningfully bias comparisons of hatchability of fertile eggs, which are based on the fertile subset within each group.

**Figure 1:**
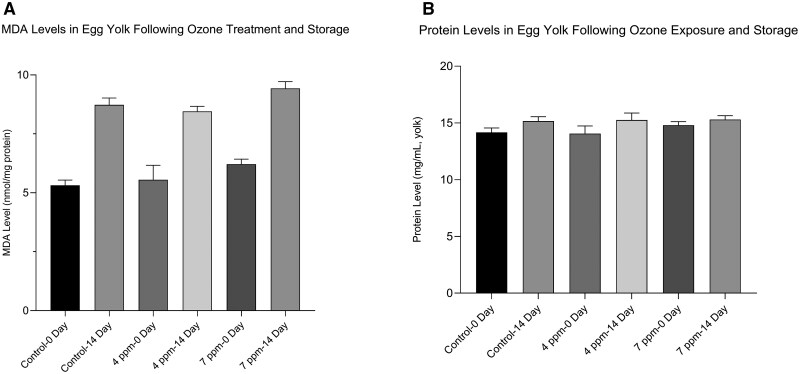
Effects of ozone dose (0, 4, and 7 ppm) and storage duration (0 and 14 d) on (A) protein-normalized yolk MDA concentration (nmol/mg protein) and (B) yolk protein concentration (mg/mL). MDA was measured using the TBARS method at 532 nm, and protein concentration was determined using the Bradford assay. Bars represent mean ± SE. Data were analyzed by two-way ANOVA (storage duration × ozone dose) followed by Tukey’s multiple comparisons test (*P* < 0.05). The greatest MDA concentration was observed in the 7 ppm–14 d group. A significant storage duration × ozone dose interaction was observed for MDA levels (*P* < 0.05).

The MDA level—an indicator of lipid peroxidation in egg yolk—was affected very significantly by both storage duration and ozone dose levels (*P* < 0.001). As storage duration increased, MDA levels also increased. At both storage durations, the 7 ppm ozone group exhibited higher MDA values than the other groups (P ≤ 0.001) (Table 3). The protein content was also affected (*P* < 0.001) by storage duration, increasing overall from 14.34 mg/mL to 15.24 mg/mL (Figure 1).

**Table 3:** Egg yolk malondialdehyde (MDA) levels (nmol/mg protein) and protein content, mg/mL, yolk.

Storage	Application	MDA, nmol/mg protein	Protein content, mg/mL
	0 ppm	5.321 ± 0.03^b^	14.16 ± 0.05
**0**	4 ppm	5.553 ± 0.09^b^	14.06 ± 0.09
	7 ppm	6.213 ± 0.03^a^	14.80 ± 0.04
***P*-value**		< 0.001	0.831
**14**	0 ppm	8.733 ± 0.04^b^	15.16 ± 0.05^b^
	4 ppm	8.453 ± 0.03^b^	15.25 ± 0.08^b^
	7 ppm	9.429 ± 0.04^a^	15.31 ± 0.04^a^
***P*-value**		0.002	0.022
**Total**			
** 0**		5.695 ± 0.03^b^	14.34 ± 0.08^b^
** 14**		8.871 ± 0.02^a^	15.24 ± 0.06^a^
	0 ppm	7.026 ± 0.13^b^	14.66 ± 0.05^b^
	4 ppm	7.002 ± 0.11^b^	14.65 ± 0.12^b^
	7 ppm	7.821 ± 0.12^a^	15.05 ± 0.06^a^
**Storage**		<0.001	<0.01
**Application**		<0.001	0.051
**Storage × Application**		0.117	0.114

Different letters (a, b) in the same column indicate statistically significant differences (*P* < 0.05). SE: standard error.

When the data on egg quality traits were evaluated, storage duration was found to have a statistically significant effect (*P* < 0.001) on all variables except egg weight, shape index, and shell percentage (ie albumen percentage, yolk percentage, albumen and yolk index values, pH, and Haugh unit) (Table 4). In particular, albumen quality indicators (albumen percentage, albumen index, and Haugh unit) deteriorated with storage, while yolk index and pH values also changed. The disinfection doses applied did not have a significant effect on the parameters examined, and no interaction was observed between storage duration and treatment (*P* > 0.05) (Table 4).

**Table 4:** Egg quality characteristics.

Storage	Application	Egg	Shape index (%)	Albumen	Yolk	Albumen	Yolk	Shell	pH	Shell	HU (%)
		weight (g)		ratio (%)	ratio (%)	index (%)	index (%)	ratio (%)		thickness (%)	
	0 ppm	71.53 ± 0.49	78.89 ± 0.54	57.69 ± 0.28	31.47 ± 0.19	11.69 ± 0.36	42.34 ± 0.51	10.84 ± 0.12	8.33 ± 0.04	0.39 ± 0.01	89.84 ± 1.27
**0**	4 ppm	70.18 ± 0.53	78.03 ± 0.30	57.17 ± 0.39	31.96 ± 0.33	10.55 ± 0.15	40.45 ± 0.77	10.87 ± 0.07	8.49 ± 0.08	0.38 ± 0.01	87.32 ± 0.54
	7 ppm	70.17 ± 0.62	77.41 ± 0.23	58.63 ± 0.41	30.47 ± 0.41	10.76 ± 0.16	42.11 ± 1.02	10.91 ± 0.09	8.63 ± 0.08	0.39 ± 0.00	87.14 ± 0.57
	0 ppm	69.08 ± 0.40	78.06 ± 0.32	53.86 ± 0.20	35.33 ± 0.22	6.26 ± 0.15	34.10 ± 0.36	10.81 ± 0.08	9.40 ± 0.01	0.39 ± 0.01	67.92 ± 1.03
**14**	4 ppm	68.21 ± 0.38	80.56 ± 0.47	54.10 ± 0.22	34.68 ± 0.46	6.26 ± 0.25	34.40 ± 0.57	11.22 ± 0.10	9.36 ± 0.01	0.39 ± 0.01	71.72 ± 2.01
	7 ppm	69.13 ± 0.35	78.59 ± 0.28	52.12 ± 0.55	36.74 ± 0.17	5.44 ± 0.14	31.47 ± 0.87	11.14 ± 0.11	9.35 ± 0.00	0.40 ± 0.01	67.08 ± 0.49
**Total**											
**0**		70.62 ± 0.17	78.40 ± 0.12	57.82 ± 0.12	31.30 ± 0.10	10.99 ± 0.08	41.63 ± 0.26	10.87 ± 0.03	8.48 ± 0.02	0.39 ± 0.00	88.10 ± 0.29
**14**		68.85 ± 0.12	77.79 ± 0.13	53.36 ± 0.13	35.58 ± 0.11	5.98 ± 0.06	33.32 ± 0.21	11.05 ± 0.03	9.37 ± 0.00	0.40 ± 0.00	68.90 ± 0.42
	0 ppm	70.62 ± 0.23	78.11 ± 0.21	55.77 ± 0.19	33.39 ± 0.21	8.97 ± 0.24	38.22 ± 0.37	10.82 ± 0.05	8.86 ± 0.04	0.39 ± 0.00	78.88 ± 0.29
	4 ppm	70.16 ± 0.23	78.49 ± 0.21	55.63 ± 0.25	33.31 ± 0.23	8.40 ± 0.19	37.42 ± 0.39	11.04 ± 0.04	8.92 ± 0.04	0.39 ± 0.00	79.52 ± 0.37
	7 ppm	68.80 ± 0.24	79.07 ± 0.13	55.37 ± 0.35	33.60 ± 0.28	8.10 ± 0.21	36.79 ± 0.60	11.02 ± 0.05	8.99 ± 0.04	0.39 ± 0.00	77.11 ± 0.42
***P* (value)**											
** Storage**		0.082	0.224	0.000	0.000	0.000	0.000	0.377	0.000	0.478	0.000
** Application**		0.671	0.424	0.891	0.941	0.320	0.757	0.642	0.615	0.975	0.443
** Storage × Application**		0.850	0.246	0.202	0.183	0.562	0.494	0.738	0.432	0.803	0.873

Different letters (a, b) in the same column indicate statistically significant differences (*P* < 0.05). SE: standard error. Minor deviations from 100% are due to rounding.

## Discussion

The present study jointly evaluated the effects of ozone applied at different doses (0, 4, 7 ppm) during long-term storage (14 d) of eggs obtained from 67-wk-old Ross 308 breeders on hatchery performance, eggshell microbial load, egg quality, and egg yolk composition (protein content and oxidative stability).

In the poultry sector, various researchers indicate that, to achieve optimal hatchery efficiency, storing hatching eggs under appropriate environmental conditions for approximately 7–10 d is ideal (Fasenko 2007). Longer storage has been shown to cause substantial changes in organoleptic, physicochemical, and microbiological properties of eggs (Nasri et al. 2020; Tabib et al. 2021). In the present research, consistent with previous reports, storage for 14 d did not affect shell quality or shape index (Tabib et al. 2021; Biesek et al. 2024) but decreased albumen percentage and albumen index (Abioja et al. 2021; Tabib et al. 2021), increased albumen pH (Nasri et al. 2020; Tabib et al. 2021; Biesek et al. 2024; Özbaşer Bulut et al. 2025), decreased yolk percentage and yolk index (Abioja et al. 2021), and reduced the Haugh unit, an indicator of egg freshness (Tabib et al. 2021; Özbaşer Bulut et al. 2025). In long-stored eggs, the dissociation of carbonic acid in thick albumen and the escape of CO_2_ through pores, the change (increase) in albumen pH, weakening of the ovomucin layer responsible for the firmness of the thick albumen, and weakening of the vitelline membrane surrounding the yolk may occur. These changes may lead to weight loss in the egg, a decrease in albumen height, an increase in albumen length and width, and, due to liquefaction of the albumen failing to support the yolk, a decrease in yolk height (Jadhav et al. 2007; Kralik et al. 2014; Lall et al. 2018; Alshaikhi et al. 2021).

As a strong oxidant, ozone is used in poultry production primarily for sanitation-oriented microbial decontamination and has benefits in contributing to food safety in production environments. Studies show that ozone can effectively disinfect eggshells, significantly reducing microbial load (Clímaco et al. 2018; Cano et al. 2019; Yuceer and Caner 2020; Silva Soares et al. 2022). Aganhar Parvin et al. (2020) reported that applying 6 and 10 ppm ozone to the eggshell completely inactivated Salmonella enteritidis; Voloshin (2016) reported a 99.89% reduction in total bacterial contamination after ozone treatment; and Timchenko et al. (2024) reported 30% and 40% reductions in microbial load following double and triple ozone applications, respectively. In line with these results, the present study determined that applying ozone at 4 and 7 ppm inhibited colony growth on eggshells, whereas significant growth occurred in the control group compared with other groups. Examinations suggest that ozone applied at different doses during long-term storage did not, by itself, produce a significant ameliorative or preventive effect on the physicochemical deterioration parameters observed in egg quality. Taken together, these findings support the view that ozone primarily acts as a sanitizing agent that suppresses microbial contamination and helps preserve internal egg quality; when applied at an appropriately optimized low dose (4 ppm), this preservation of microbial and oxidative status appears to contribute to a more favorable embryonic environment and improved hatchability, whereas higher doses may disturb this balance and exert embryotoxic effects (Fuhrmann et al. 2010; Aganhar Parvin et al. 2020). Yüceer et al. (2016) reported that, in eggs stored for 14 d, a single short application (2–5 min) at similar doses (4 and 6 ppm) preserved the Haugh unit, increased the resilience of the vitelline membrane, and slowed water transfer, and slowed CO_2_ loss to prevent the increase in albumen pH. Perry and Yousef (2013) stated that ozone applications combined with heat treatment could preserve egg quality during storage and yield positive effects on the Haugh unit and yolk index. These observations suggest that differences in application mode or storage conditions may influence outcomes. Further studies on application modalities are planned.

In hatchery operations, parameters such as hatchability and hatch of fertile eggs are important indicators affecting profitability. They reflect not only incubator performance but also the quality of the entire production chain, from breeder flock management to egg collection and storage. Hatching outcomes may be influenced by genotype, breeder age, nutritional status, storage conditions, and disinfection procedures applied to eggs (Fasenko 2007). As is generally accepted, long-term storage reduces egg quality and negatively affects hatching outcomes. In the present study, applying 4 ppm ozone to stored eggs mitigated the adverse effects of storage and significantly increased hatch of fertile eggs and hatchability. This finding is consistent with some reports in the literature. Hrnčár et al. (2021) reported that a lower dose (0.45 ppm) but longer exposure (12 h) similarly improved hatch and hatchability while reducing embryonic mortality, suggesting that low-to-moderate ozone applications may support embryonic viability. However, the effectiveness of ozone appears to be highly dependent on parameters such as dose and duration. Indeed, Wlazlo et al. (2020) reported that, in quail eggs, a high dose (4.2 mg/h) and short exposure (5 min) decreased hatchability and increased embryonic deaths. This contradictory result indicates that high ozone doses may exert toxic effects and that species-specific responses may differ. The favorable outcome of 4 ppm in the present study suggests that this dose falls within an “optimal” range for broiler eggs. On the other hand, some studies (Koç and Aygün 2021; Yılmaz Dikmen et al. 2025) reported that ozone application did not produce statistically significant effects on traditional hatchery performance parameters (hatchability, embryonic mortality). Although these findings do not fully align with ours, they underscore one of the most consistent benefits of ozone: reducing microbial load. This may be one of the primary mechanisms underlying the improved hatch performance observed in our study; in stored eggs, the increased microbial burden may have been controlled by ozone, thereby providing a healthier environment for embryonic development. Moreover, some researchers have noted that, in addition to reducing microbial load, ozone disinfection—as in our study—may also shorten hatch time (Yılmaz Dikmen et al. 2025), highlighting another noteworthy potential benefit of ozone application. The shorter incubation length observed in the 4 ppm group may reflect improved embryonic development dynamics, potentially associated with reduced microbial load and a more favorable egg microenvironment. This observation supports the hypothesis that optimized low-dose ozone exposure may positively influence embryonic development conditions.

MDA, one of the markers of lipid peroxidation, is a parameter that helps objectively and quantitatively determine the level of oxidative deterioration in eggs (Dotas et al. 2023). Egg yolk is rich in unsaturated fatty acids (particularly polyunsaturated fatty acids, PUFAs) and phospholipids. When exposed to factors such as oxygen, heat, light, and metal ions, these components undergo oxidation. MDA, a secondary product of this oxidation process, is used as a reliable indicator of the extent of oxidation (Chen et al. 2022). In our study, determining MDA levels in egg yolk served as a quantitative indicator of oxidative stress induced by storage and ozone application. The findings clearly revealed the adverse effects of long-term storage on egg quality. The marked increase in MDA after 14 d of storage (d 0: 5.695 nmol/mg protein; d 14: 8.871 nmol/mg protein) corroborates that, as storage lengthens, oxygen ingress into the egg increases, free-radical chain reactions accelerate, natural antioxidants are depleted, and lipid peroxidation progresses (Ren et al. 2013). This is consistent with the findings of Mierliţă (2019), who reported elevated MDA levels with longer storage durations. These lipid peroxidation data help to elucidate the mechanism underlying the embryotoxic effect of the high ozone dose, indicating that excessive oxidative damage is associated with reduced hatchability. In contrast, our findings demonstrate a clear dose-dependent response: the 7 ppm treatment was accompanied by elevated yolk MDA levels and impaired hatching performance, whereas 4 ppm ozone-maintained yolk oxidative status, reduced eggshell microbial load, and was associated with improved hatchability of fertile eggs and lower early embryonic mortality. This suggests that appropriately optimized low-dose ozone does not merely act as a surface disinfectant but may also promote a more favorable embryonic environment by limiting microbial challenge and preserving yolk lipid integrity, thereby supporting embryonic development (Saad 2024; Yılmaz Dikmen et al. 2025). In contrast, a higher dose of 7 ppm may have exacerbated the oxidative milieu induced by storage, triggering lipid peroxidation (increased MDA) and causing undesirable changes in protein structures. The early stages of embryonic development rely on lipid and protein resources that are vital for the synthesis of cell membranes and structural proteins (Zhu et al. 2020). Therefore, this combined lipid and protein damage caused by a high ozone dose is considered the principal reason for the high early embryonic mortality (11.76%) observed in the 7 ppm group. Mattioli et al. (2020) also reported increases in lipid oxidation products following ozone applications at a flow rate of 600 mg/h for 2 h, supporting this mechanism. In sum, these findings support the view that ozone at low concentrations may be adaptive and protective, whereas at high concentrations it is prone to induce oxidative damage.

In the present study, yolk protein concentration increased progressively with storage duration, which can be attributed to moisture loss and water redistribution between albumen and yolk, leading to a relative concentration of yolk solids (Samli et al. 2005). High-dose ozone treatment (7 ppm) resulted in a numerically higher yolk protein content at d 0 compared with the control (14.80 vs. 14.16 mg/mL), although this difference was not statistically significant. This trend may indicate subtle changes in protein solubility potentially associated with oxidative modification at high ozone levels, consistent with reports that strong oxidizing treatments can alter egg protein structure and functionality (Uzun et al. 2012). However, the 4 ppm treatment did not adversely affect yolk protein stability and was associated with improved fertility and reduced embryonic mortality, suggesting that appropriately optimized low-dose ozone can be used without detrimental effects on yolk protein integrity under the conditions of this study.

## Conclusion

In summary, daily application of low-dose ozone (4 ppm) during storage of broiler hatching eggs reduced eggshell microbial load and partially counteracted the negative impact of prolonged storage on hatchability without inducing a marked increase in yolk lipid peroxidation. In contrast, the higher ozone dose (7 ppm) was associated with elevated yolk MDA concentrations and greater early embryonic mortality, indicating that oxidative stress outweighed any sanitary benefit at this level. From a practical standpoint, these findings suggest that controlled, low-dose ozone exposure can be integrated into hatchery management as a supportive strategy to maintain egg quality and promote embryonic viability and hatchability in long-stored eggs, whereas higher ozone levels should be avoided because of their potential to disrupt oxidative balance and compromise hatching performance.

## Data Availability

The datasets generated and analyzed during the current study are available from the corresponding author on reasonable request.

## Abbreviations

ANOVA: analysis of variance
CFU: colony-forming unit
HU: Haugh unit
MDA: malondialdehyde
PBS: phosphate-buffered saline
ppm: parts per million
SE: standard error
TBARS: thiobarbituric acid reactive substances.
